# Effect of Freeze–Thaw Cycles on the Shear Strength of Root-Soil Composite

**DOI:** 10.3390/ma17020285

**Published:** 2024-01-05

**Authors:** Qi Liu, Jiankun Huang, Zhiwei Zhang, Gongming Liu, Qunou Jiang, Lanhua Liu, Inam Khan

**Affiliations:** 1 Key Laboratory of State Forestry Administration on Soil and Water Conservation, School of Soil and Water Conservation, Beijing Forestry University, Beijing 100083, China; 18801083792@163.com (Q.L.);; 2 Three-Gorges Reservoir Area (Chongqing) Forest Ecosystem Research Station, School of Soil and Water Conservation, Beijing Forestry University, Beijing 100083, China; 3 School of Soil and Water Conservation, Beijing Forestry University, Beijing 100083, China; 4 Energy Saving & Environmental Protection & Occupational Safety and Health Research Institute, China Academy of Railway Science Co., Ltd., Beijing 100081, China; 5 College of Forestry, Beijing Forestry University, Beijing 100083, China; inamkhan.1325@gmail.com

**Keywords:** root-soil composite, freeze–thaw cycle, shear strength, direct shear test, numerical simulation

## Abstract

A large alpine meadow in a seasonal permafrost zone exists in the west of Sichuan, which belongs to a part of the Qinghai–Tibet Plateau, China. Due to the extreme climates and repeated freeze–thaw cycling, resulting in a diminishment in soil shear strength, disasters occur frequently. Plant roots increase the complexity of the soil freeze–thaw strength problem. This study applied the freeze–thaw cycle and direct shear tests to investigate the change in the shear strength of root-soil composite under freeze–thaw cycles. This study examined how freeze–thaw cycles and initial moisture content affect the shear strength of two sorts of soil: uncovered soil and root-soil composite. By analyzing the test information, the analysts created numerical conditions to foresee the shear quality of both sorts of soil under shifting freeze–thaw times and starting moisture levels. The results showed that: (1) Compared to the bare soil, the root-soil composite was less affected by freeze–thaw cycles in the early stage, and the shear strength of both sorts of soil was stabilized after 3–5 freeze–thaw cycles. (2) The cohesion of bare soil decreased more than that of root-soil composite with increasing moisture content. However, freeze–thaw cycles primarily influence soil cohesion more than the internal friction angle. The cohesion modification leads to changes in shear quality for both uncovered soil and root-soil composite. (3) The fitting equations obtained via experiments were used to simulate direct shear tests. The numerical results are compared with the experimental data. The difference in the soil cohesion and root-soil composite cohesion between the experiment data and the simulated result is 8.2% and 17.2%, respectively, which indicates the feasibility of the fitting equations applied to the numerical simulation of the soil and root-soil composite under the freeze–thaw process. The findings give potential applications on engineering and disaster prevention in alpine regions.

## 1. Introduction

A large alpine meadow in a seasonal permafrost zone is widely distributed in western Sichuan, which belongs to a part of the Qinghai–Tibet Plateau, China. The freeze–thaw cycle, which is caused by the large diurnal temperature variation and climate change, seriously affects the engineering properties of slopes. However, the root system of herbaceous plants is slim. The shear strength of root-soil composite will be significantly affected under the freeze–thaw cycle, and the slope will be easy to slide under the deterioration of repeated freeze–thaw damage due to temperature variation. Meanwhile, soil moisture changes from outside variables (rainfall, snow melt, etc.) essentially influence slope stability and mechanical properties. Observing and understanding these variances are imperative for evaluating and mitigating slope hazards. A series of special phenomena, such as freeze swelling, thawing, and strength weakening, have multiple impacts on the construction and project operation [1,2]. Therefore, understanding the shear strength of bare soil and root-soil composite under freeze–thaw cycles is a core research topic that has attracted interest in recent years.

Freeze–thaw cycles change the mechanical properties and structural characteristics of soils [3,4,5,6,7] and lead to soil deformation. A study on the physical properties of soils amid freeze–thaw cycles fundamentally emphasized the impact of two variables: the number of freeze–thaw cycles and the initial moisture content on soil shear strength. The studies uncovered that the soil’s strength and cohesion declined when subjected to freeze–thaw cycles, while the soil’s internal friction angle increased exponentially [8,9,10]. The first freeze–thaw cycle leads the most obvious reduction in the internal friction angle and cohesion during the entire freeze–thaw cycle. The internal friction angle and cohesion gradually stabilized after the 5th–7th freeze–thaw cycles [11]. Soil strength decreased with the increasing number of freeze–thaw cycles and soil moisture content [12,13].

Previous research has fundamentally concentrated on analyzing how freeze–thaw cycles affect the shear strength of bare soils, with restricted studies on the freeze–thaw impacts on the strength of root-soil composites. However, in later times, the reinforcement given by vegetation roots has picked up critical consideration and interest among analysts and experts. Understanding how freeze–thaw activities influence the strength of root-soil composites is fundamental for comprehending the role of vegetation in slope stability and soil mechanics. Plant roots improve soil shear strength and enhance the resistance of volume deformation, which increases slope stability [14,15,16,17]. Vegetation roots also influence the freeze–thaw cycle process by modifying the physical properties of the soil and its intrinsic root morphology characteristics. The existence of roots changes the soil moisture content and suction, thus reducing the appearance of soil thawing, sinking, and cracking [18]. Plant roots play a reinforcement effect on slopes even though under the freeze–thaw action [19,20]. But inconsistent conclusions were obtained due to the different types of soils studied in variable field conditions [21,22]. However, previous studies have investigated the reinforcing impact of plant roots on soil shear strength amid freeze–thaw conditions, and they have not broadly considered the combined effect of different components. The disaster-causing mechanism has long remained unclear, mainly because of the complexity of root-soil composite, which is a type of bioengineering methodology [23]. Particularly, the complicated coupling of heat–moisture–stress fields as well as the interaction between introductory soil moisture content, root substance, and the number of freeze–thaw cycles on soil strength have not been completely explored. Understanding these complex interactions is significant for a comprehensive understanding of how plant roots contribute to soil stability under changing freeze–thaw conditions. This poses great challenges to potential applications on engineering and disaster prevention in alpine regions.

In this paper, herbaceous vegetated slopes in west Sichuan were studied by analyzing the effects of initial moisture content, root content, and the number of freeze–thaw cycles on the shear strength of root-soil composite under freeze–thaw cycle conditions. The structure of the remaining paper is as follows. A combination of freeze–thaw cycle tests and direct shear tests was utilized. Moreover, the research investigated the strength changes in both uncovered soil and root-soil composite under different influencing factors. These components might include changing freeze–thaw cycle conditions, diverse initial moisture content, and the presence of plant roots inside the soil. The objective was to determine how each calculation contributes to the differences in shear strength behavior observed between uncovered soil and root-soil composite amid freeze–thaw cycles. Secondly, the fitting equations of the coupling effect of soil moisture content, root content, and number of freeze–thaw cycles on soil shear strength under freeze–thaw cycles were obtained using theoretical analysis. Finally, the fitting equations were applied to numerical simulations. The feasibility and accuracy of the theoretical equations applied to numerical simulations were validated.

## 2. Materials and Methods

### 2.1. Study Site

The area of seasonally frozen soil areas in China is approximately 5.137 million km^2^, accounting for 53.5% of the national land area. The study area is located in western Sichuan, which is also on the eastern edge of the Qinghai–Tibet Plateau, with a typical seasonal frozen soil area. This area has plateau climate characteristics, sufficient sunshine, and a large temperature difference between day and night. The average annual temperature is 7.1 °C during 2011–2021. The annual precipitation of the study area is 800–950 mm. There is no summer, but long snow and ice periods. The soil type is subalpine meadow soil with 33.31% vegetation cover. The herbaceous vegetation in this area is mainly *Astragalus sinicus* L. and *Pyrolaceae*.

### 2.2. Experimental Materials

#### 2.2.1. Material Parameters

**Figure 1:**
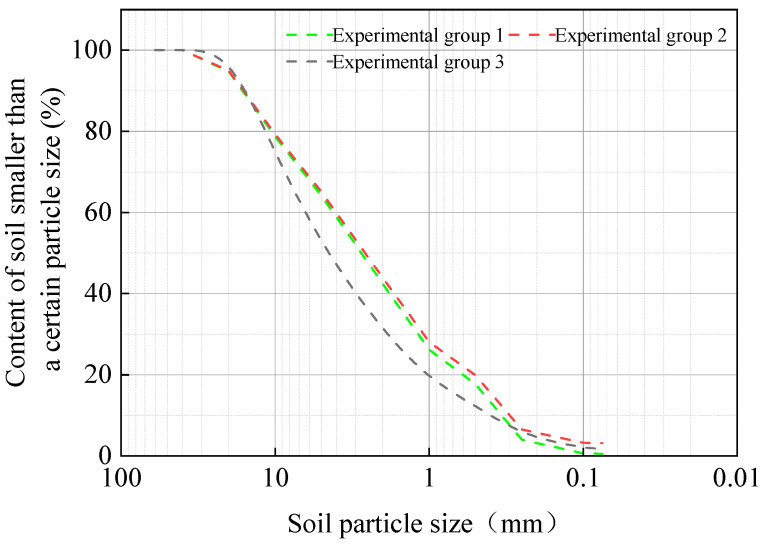
Particle gradation curve of bare soil.

This study selected undisturbed root-soil composite samples and disturbed uncovered slope soil samples from an engineering development location. On-site sampling was performed utilizing ring knives to obtain representative soil samples. To survey the fundamental physical properties of the soil, indoor drying and sieving tests were conducted on the collected samples. These tests were used to analyze key soil characteristics and provide valuable information for their study as shown in Table 1. The particle gradation curve of the bare soil is shown in Figure 1.

#### 2.2.2. Specimen Preparation Method

**Figure 2:**
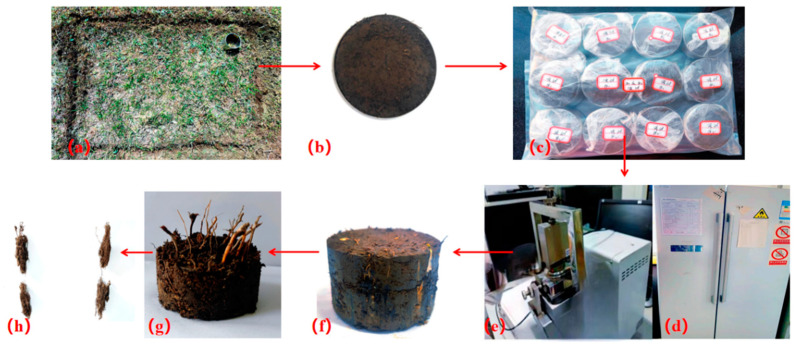
Sampling and test process. (**a**) A plot of root-soil composite, (**b**) ring knife sampling, (**c**) maintaining the moisture content via sealed samples, (**d**) a freeze–thaw cycle device, (**e**) the direct shear test, (**f**) sample after shearing, (**g**) root distribution of sample, and (**h**) calculating root content.

Figure 2 illustrates the method of sampling, sample planning, and testing for this study. The roots of the vegetation were found to be densely dispersed within the selected area. Before conducting the direct shear test, three particular plots on the slope were chosen, each representing different plant growth conditions. It was ensured that plant growth was reliable inside each plot. In each plot, in situ root-soil composite samples (Figure 2a) were collected using ring knives and then transported to the laboratory for further analysis. Upon collection, the vegetation roots within the ring knives were carefully washed, dried, and weighed. Any data with excessively large or low root weights, which could skew the results, were prohibited from the analysis. This thorough process ensured the exactness and reliability of the data utilized for evaluating the impact of vegetation roots on the soil’s mechanical properties. The root area ratio (RAR) method was used to determine the root content [24]. The RAR is 0.9%, 3.2%, and 7.5%, respectively, for the three in situ tests. The average minimum temperature was −7 °C in winter and the average maximum temperature was 20 °C in summer according to the local temperature data in the past 10 years. Therefore, the freezing temperature of the freeze–thaw cycle test was designed to be −10 °C and the thawing temperature was 20 °C to simulate the actual temperature of the site. Twenty-four hours was considered as a cycle period.

The 61.8 mm × 40 mm ring knife was used to sample soil in three different in situ plots. Before sampling, an undisturbed and well-grown area was selected for in situ soil sampling (Figure 2a). Sampling preparation included pressing the ring knife vertically into the soil utilizing its handle. Subsequently, any grass leaves and excessive roots were removed by cutting them with scissors. The soil samples on both sides of the ring knife were then sharpened using a soil-cutting knife (Figure 2b). To protect the natural moisture content of the samples and avoid moisture loss, the collected soil samples were wrapped in cling film (Figure 2c) instantly after collection. The wrapped samples were instantly transported back to the test room from the location to guarantee exact and dependable test results based on the natural moisture content of the soil. This careful procedure aimed to preserve the first conditions of the soil samples and avoid any potential change in their properties due to moisture changes amid transportation and testing. Three initial moisture contents were investigated accounting for the evaporation of water and melting of snow and ice in winter. According to the soil dry weight and design moisture content, the corresponding volume of distilled water was evenly dripped on the upper and lower sides of the specimen using a rubber-tipped dropper, and then wrapped in cling film and left for 12 h for full moisture diffusion. Finally, these samples were placed in a −10 °C refrigerator to start the 12 h freeze (Figure 2d). The freezing process occurs from the surface to the insides. However, for test purposes and considering the small size of the ring knife utilized, whether it adopts one-way freezing or three-way freezing does not significantly influence the results. Hence, the freezing heading was not a major concern. To simulate natural freeze–thaw conditions, the soil samples experienced a 12 h thawing period after freezing. Therefore, a total freeze–thaw cycle comprised 24 h, with both freezing and thawing phases. This cyclic process permitted analysts to reproduce the freeze–thaw conditions that exist in natural situations and analyze and compare effects on soil strength and behavior. After finishing the designed number of freeze–thaw cycles, the direct shear test was conducted immediately (Figure 2e,f). The roots were removed and the root content was calibrated when finishing the direct shear test, as shown in Figure 2g,h.

Bare soil specimens were taken from the area surrounding the root-soil composite. The designed initial moisture content of the bare soil specimens was slightly different from that of the root-soil composite. The natural moisture content was determined through drying. Distilled water was utilized to make three distinctive initial moisture levels. The test method for both uncovered soils and root-soil composite was the same, ensuring consistent procedures for accurate comparison.

### 2.3. The Direct Shear Test Method under Freeze–Thaw Cycles

The test was set with three types of initial moisture content. The previous studies showed that soil shear strength gradually stabilized if the number of freeze–thaw cycles was up to five. Therefore, four types of cycles, i.e., 0, 1, 3, and 5 freeze–thaw cycles, were used in the test. The experimental design is shown in Table 2.

The direct shear test was conducted via the automatic strain-controlled direct shear instrument manufactured by Huakan Technology Co. (Beijing, China). The soil specimen completed the specified number of freeze–thaw cycles, it was set into the coordinate shear instrument for testing. The shear rate was set at 0.8 mm/min.

During the test, the specimen was subjected to different vertical weights of 100, 200, 300, and 400 kPa, respectively, to analyze the shear strength under shifting stress conditions. After each test was completed, the shear instrument was reset for the next trial. The data obtained from the tests were transmitted to a computer terminal through a data procurement device, enabling exact data recording and analysis. We compared *Geotechnical Test Method Standard GB/T 50123-2019* [25] and *Geotechnical Investigation and Testing-Laboratory Testing of Soil*, *ISO 17892-10:2018* [26]. The two standards have a slight difference in the calculation of soil cohesion. The calculation method of the former is more consistent with the experimental instruments in China. Therefore, *Geotechnical Test Method Standard GB/T 50123-2019* was used. The peak or stable value on the relationship curve between shear stress, *τ*, and shear displacement, Δ*L*, is selected as the shear strength. If there is no peak, the shear stress corresponding to shear displacement, Δ*L* = 4 mm, is taken as the shear strength. Then, the *c* and *φ* values of the tested samples were obtained.

### 2.4. The Numerical Simulation Method

In order to observe the stress changes in soil after freeze–thaw cycles using the direct shear tests and analyzing the mechanism of freeze–thaw cycles, numerical simulation was used to compare and analyze the results of the direct shear test under freeze–thaw cycles. Meanwhile, the fitting equations obtained from the above experiments were applied to numerical simulations, which can validate the applicability of the fitting equations.

#### 2.4.1. Bare Soil Modeling and Boundary Conditions

**Figure 3:**
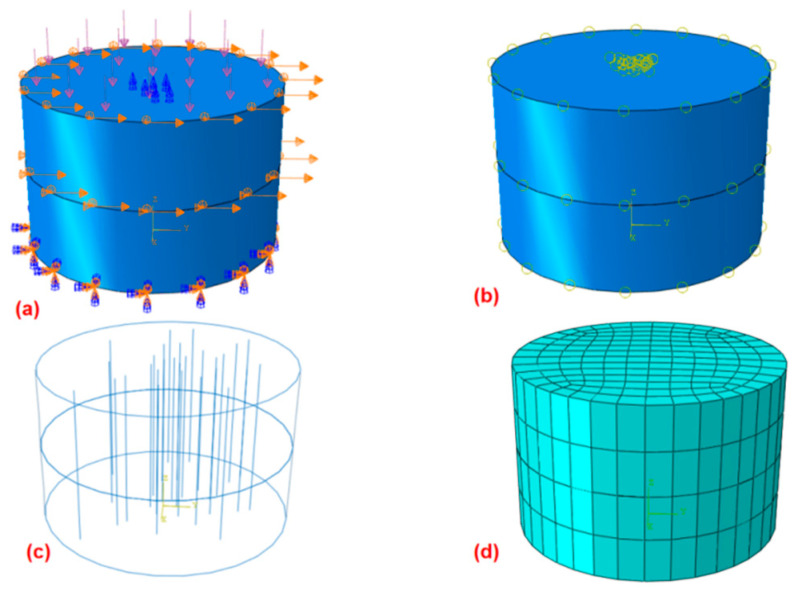
Boundary conditions and meshes of the simulation model. (**a**) Boundary conditions, (**b**) roots with built-in area, (**c**) sample with random root distribution, and (**d**) finite element meshes.

The numerical simulation model adjusts accurately with the geometry of the direct shear test in finite element software ABAQUS 6.20-1. The shear box utilized within the simulation is cylindrical and has been divided into two sections. The radius of the cylinder measures *r =* 30.9 mm, while its height stands at *h* = 40 mm. The boundary conditions utilized within the numerical simulation reflect those utilized within the direct shear test. The bottom of the lower box is constrained in the U1, U2, and U3 (i.e., X-, Y-, Z-) directional displacements as well as the relative rotations during the pressurization. The upper and lower boxes were constrained to the displacement in the U1 and U2 (i.e., X- and Y-) directions. The top of the upper box was added a uniform load. The rest parts are free boundaries. The upper box is modified to produce a shear displacement of 6 mm in U2 (i.e., Y-) direction as the shear motion. This is consistent with the direct shear test, as shown in Figure 3a,b. The interaction between the upper and lower shear boxes amid soil shearing is modeled as a contact type characterized by an identical friction coefficient. This coefficient is known as the Mohr–Coulomb criterion. The cohesion is approximated by calculating the friction coefficient with the varied pressure as Equations (1) and (2).

(1) $$\tau =c+\mu \sigma =\mu^{*}\sigma$$

(2) $$\mu^{*}=\left(c+\mu \sigma \right)/\sigma =\mu +c/\sigma$$

where *τ* is soil shear strength; *c* is the soil cohesion; *μ* is the initial friction coefficient of the soil; *σ* is the vertical pressure; and μ* is the equivalent friction coefficient.

#### 2.4.2. Root-Soil Composite Modeling and Boundary Conditions

For the root-soil simulation, only the high root content (i.e., RAR = 7.5%) was used as the simulation object. The root distribution was simulated by MATLAB 2020a program algorithm. The frequency function of log-normal distribution was used to simulate the random distribution characteristics of the roots [27]. In ABAQUS, the root distribution model was coordinated, centering solely on the vertical arrangement of roots, as the herbaceous roots’ morphology basically impacts these coordinates. A truss element was utilized to represent the roots within the simulation. The roots were implanted inside the soil, and a predefined area was executed to account for the interaction between the roots and the soil. Furthermore, to prevent any rotational movement, the bottom of the roots was constrained in UR3. The boundary conditions of the soil are the same as the bare soil (as shown in Figure 3a,b). Since the roots of the vegetation in the experimental area are mainly dominated by primary roots and without excessive lateral roots, as shown in Figure 2g,h, the root model is simplified as shown in Figure 3c.

#### 2.4.3. Parameter and Mesh of Simulation Models

The mechanical behavior of the soil was modeled utilizing the well-established Mohr–Coulomb constitutive model, which is competent for describing both elastic and plastic deformation characteristics. To simulate the different soil conditions, two scenarios were considered: uncovered soil with a moisture content of 32% and a root-soil composite with 26% moisture substance after undergoing a freeze–thaw cycle. The important parameters corresponding to each condition were consolidated into the model for accurate representation and analysis. The soil elastic modulus refers to soft clay, which is taken as 4 MPa. Poisson’s ratio is taken as 0.35. The root elastic modulus refers to herbaceous roots, and is taken as 2 MPa. The root Poisson ratio is taken as 0.2. The influence of freeze–thaw cycles on the soil parameters, *c* and *φ*, was considered based on the freeze–thaw cycle test, which is defined in Section 3.4. The meshes of the element model were shown in Figure 3d.

## 3. Results

### 3.1. Effect of Initial Moisture Content on the Shear Strength of Specimens under Freezing and Thawing

#### 3.1.1. Bare Soil Case

**Figure 4:**
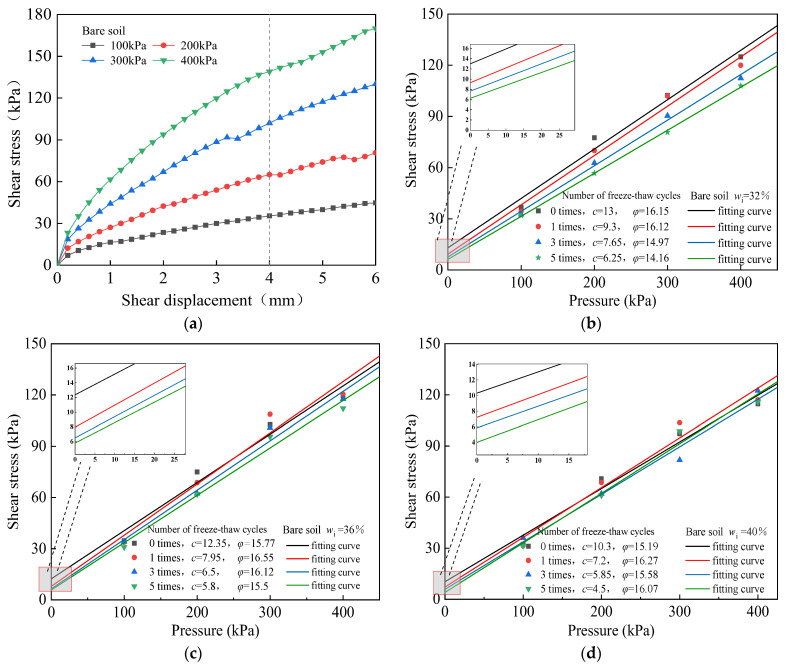
Fitting curve of the shear strength of bare soil with different initial moisture content under freeze–thaw cycles. (**a**) Stress–displacement curve of bare soil with *w*_i_ = 32% and no freeze–thaw, fitting curves of the freeze–thaw shear strength of bare soil with (**b**) *w*_i_ = 32%, (**c**) *w*_i_ = 36%, (**d**) *w*_i_ = 40%, respectively.

As an illustrative example, data from a group of uncovered soils with an initial moisture content of *w*_i_ = 32% were delineated in Figure 4a. Subsequently, shear strength curves for the bare soil subjected to freeze–thaw cycles were fitted and shown in Figure 4b–d, individually. These figures provide valuable insights into the variations in shear strength under diverse freeze–thaw conditions for the bare soil samples. The formula for calculating soil shear strength was adopted as: τ=c+σtanφ, in which, *φ* is the internal friction angle of soil.

After five freeze–thaw cycles, the shear strength of the bare soil with *w*_i_ = 32%, 36%, and 40%, for example, with a vertical pressure of 100 kPa, decreased by 25.6%, 16.2%, and 12.9%, respectively. The reduction in the shear strength among samples with diverse initial moisture content shows slight variance. As the initial soil moisture content increases, the percentage decrease in soil shear strength slowly decreases after experiencing freeze–thaw cycles. Furthermore, for samples with the same moisture content, the soil cohesion decreases with an increment in the number of freeze–thaw cycles. Notably, higher moisture content in the soil tends to mitigate the negative effects of freeze–thaw cycles on its strength. After five freeze–thaw cycles, the soil cohesion, *c*, decreased by 51.9%, 53%, and 56.3% for the bare soil with *w*_i_ = 32%, 36%, and 40%, respectively. The decrease in soil cohesion, *c*, gradually increased with the increase in moisture content [28,29,30,31]. The change in the soil internal friction angle, *φ*, by freeze–thaw cycles was not obvious.

#### 3.1.2. Root-Soil Composite Case

**Figure 5:**
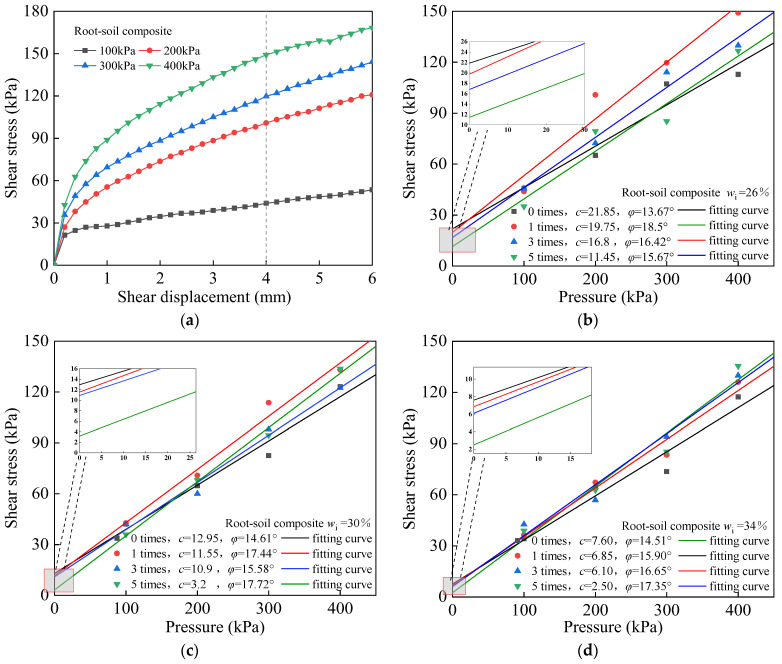
Fitting curve of the shear strength of root-soil composite under different freeze–thaw cycles. (**a**) Stress–displacement curve of root-soil composite with *w*_i_ = 26% and no freeze–thaw, fitting curves of the freeze–thaw shear strength of root-soil composite with (**b**) *w*_i_ = 26%, (**c**) *w*_i_ = 30%, (**d**) *w*_i_ = 34%, respectively.

Figure 5a shows the stress–displacement path generated by direct shear for root-soil composites with *w*_i_ = 26%. Figure 5b–d show the fitting curves of the soil shear strength of the root-soil composites undergoing different numbers of freeze–thaw cycles with different initial moisture contents.

After five freeze–thaw cycles, the shear strength of the root-soil composite with *w*_i_ = 26%, 30%, and 34% decreased by 6.64 kPa, 3.92 kPa, and 0.98 kPa under  σ_2_ = 100 kPa, respectively. The percentage of decrease was 14.4%, 10.4%, and 2.92%, respectively.

### 3.2. Effect of the Number of Freeze–Thaw Cycles on the Shear Strength of Specimens

#### 3.2.1. Bare Soil Case

**Figure 6:**
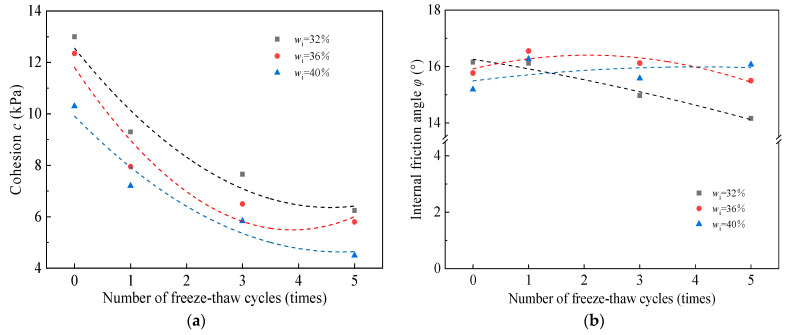
Effect of the number of freeze–thaw cycles on (**a**) *c* and (**b**) *φ* of bare soil.

Figure 6a,b show the effects of the number of freeze–thaw cycles on the cohesion, *c*, and the internal friction angle, *φ*, of the bare soil with different moisture contents. From Figure 6a, the cohesion, *c*, of the soil decreases rapidly with the increase in the number of freeze–thaw cycles. The cohesion decreases by 3.7 kPa, 4.4 kPa, and 3.1 kPa for the specimens with *w*_i_ = 32%, 36%, and 40%, respectively. After one freeze–thaw cycle, the decrease in soil cohesion accounted for 55.1%, 67.2%, and 53.4% of the decrease in the five freeze–thaw cycles, respectively.

#### 3.2.2. Root-Soil Composite Case

**Figure 7:**
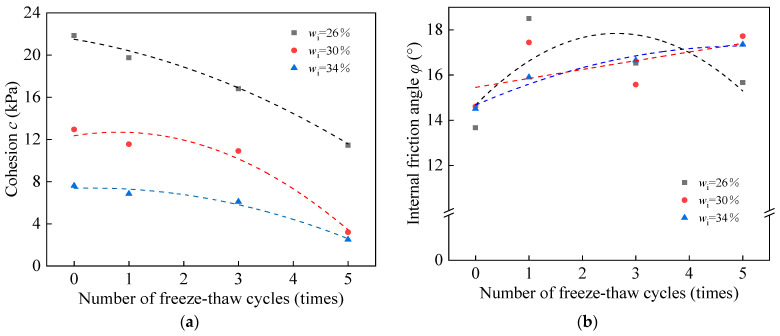
Effect of the number of freeze–thaw cycles on (**a**) *c* and (**b**) *φ* of root-soil composite.

From Figure 7a, the cohesion of the root-soil composite with shifting moisture content decreased with an increasing number of freeze–thaw cycles. However, the trend of cohesion decrease in the root-soil composite was different from that observed within the bare soil. In the early stage of freeze–thaw cycles, the cohesion decrease in the root-soil composite was generally slow; however, it became more articulated in the later stage of the cycles. The values of cohesion, *c*, decreased by 2.1 kPa, 1.4 kPa, and 0.75 kPa for specimens with *w*_i_ = 26%, 30%, and 34%, after 1st freeze–thaw cycle, respectively. The percentage of decrease was 9.6%, 10.8%, and 9.8%, respectively. From the fitting curves, the *c* value of the root-soil composite decreased more gently before three freeze–thaw cycles compared with the bare soil. During the first freeze–thaw cycle of the bare soil, a significant steep drop in cohesion was observed. The root-soil composite did not display this phenomenon. Instead, the cohesion of the root-soil composite showed a slow decrease with expanding freeze–thaw cycles; and after 3–5 cycles, the rate of decrease in the cohesion of the root-soil composite increased continuously. The cohesion of root-soil composite with *w*_i_ = 26%, 30% and 34% decreased by 5.35 kPa, 7.7 kPa and 3.6 kPa, respectively. The reduction in soil cohesion after 3–5 freeze–thaw cycles accounted for 51.4%, 78.9%, and 47.4% of the total reduction in soil cohesion after 5 freeze–thaw cycles, respectively.

### 3.3. Influence of Root Content on the Shear Strength of Root-Soil Composite during Freeze–Thaw Cycle

**Figure 8:**
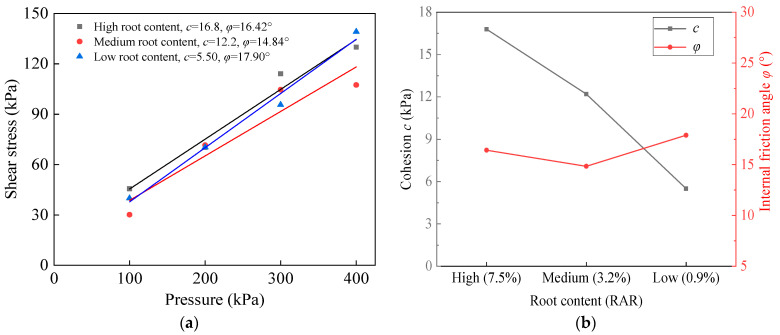
Comparison of (**a**) *c* and (**b**) *φ* of root-soil composite with different root content.

Through the control variable method, three root-soil composite specimens were chosen to represent the changing levels of root content: high, medium, and low. The point of this determination was to investigate and understand the influence of root content on the shear strength of the root-soil composite during the freeze–thaw cycles. The initial moisture content was set at *w*_i_ = 26%. The number of freeze–thaw cycles was three. Freeze–thaw cycles and direct shear tests were conducted to obtain the cohesion and internal friction angle curves as shown in Figure 8.

Under identical conditions of initial moisture content and the number of freeze–thaw cycles, the cohesion of the root-soil composite decreased relatively with a diminishment in root content. However, no significant changes were observed in the internal friction angle.

### 3.4. Theoretical Results of the Shear Strength of Samples under Freeze–Thaw Action

Taking into consideration the reinforcing impact of plant roots on soil, a mathematical formula has been derived to quantify the influence of the number of freeze–thaw cycles, initial moisture content, and root content on the shear strength of the soil. The direct shear test can be utilized to analyze the effects of the number of freeze–thaw cycles and moisture content. By utilizing this formula, the interactions between these factors and their effect on soil shear strength can be comprehensively studied and understood. In this paper, the effects of the number of freeze–thaw cycles and moisture content are added to the shear strength equation of the soil and written as:

(3) $$\tau =c+c\left(n,w_{i}\right)+\sigma \tan\left[\varphi +\varphi \left(n,w_{i}\right)\right]$$

where *n* is the number of freeze–thaw cycles. *c*(*n*, *w_i_*) and *φ*(*n*, *w_i_*) represent the effect of the number of freeze–thaw cycles and initial moisture content on the cohesion and internal friction angle of the soil, respectively.

For the root-soil composite, considering the root reinforcement effect, the root-soil composite shear strength can be expressed as:

(4) $$\tau =c+\sigma \tan\varphi +\tau_{r}$$

(5) $$\tau_{r}=\frac{\sum_{j=1}^{n_{r}}L_{j}cos(\beta_{j})}{A}+\frac{\sum_{j=1}^{n_{r}}L_{j}sin(\beta_{j})}{A}\tan\varphi$$

where *τ_r_* is the increment of the shear strength of the soil under the effect of root reinforcement; *j* denotes the *j*-th roots; *n_r_* is the total number of roots; *A* is the area of the roots acting on the soil; *β* is the shear deformation angle (i.e., 0–90°); and in this paper, for the root angle, the default is the vertical distribution with the soil. Therefore, *β* is 0°; *L* is the tensile strength of the roots. The effect of root content can be analyzed by Equation (5).

Taking into consideration the changing numbers of freeze–thaw cycles and initial moisture content, this study aims to analyze their individual impacts on the cohesion and internal friction angle of the soil. By analyzing these components, a comprehensive understanding of how soil properties change under different freeze–thaw conditions can be gained. Substituting Equation (3) into Equations (4) and (5), the shear strength of the root-soil composite considering the combined effect of root reinforcement, freeze–thaw times, and initial moisture content can be obtained:

(6) $$\tau =c+c\left(n,w\right)+\sigma \tan\left[\varphi +\varphi \left(n,w\right)\right]+\left[\frac{\sum_{j=1}^{n_{r}}L_{j}cos(\beta_{j})}{A}+\frac{\sum_{j=1}^{n_{r}}L_{j}sin(\beta_{j})}{A}\tan\varphi \right]$$

where *c* and *c*(*n*, *w*), *φ* and *φ*(*n*, *w*) are derived from the bare soil direct shear test; *L_j_* is derived from the root pullout test; *β_j_* and *A* are derived from the fast shear test of the root-soil composite; the range of values for *β_j_* is 0 to 90°. Therefore, the theoretical value of the shear strength of the root-soil composite is the range value.

Combining the above theoretical equations with the data of the direct shear tests, a quadratic polynomial for the effect of the number of freeze–thaw cycles *n* and the initial moisture content *w_i_* on the cohesion, *c*, and the internal friction angle, *φ*, is fitted for bare soil, and the cohesion *c*(*n*, *w*) can be expressed as:

(7) $$c\left(n,w\right)=11.01004-3.49756n+33.75530w+0.31537n^{2}+2.15042nw-89.84375w^{2}$$

The internal friction angle *φ*(*n*, *w*) can be expressed as:

(8) $$\varphi \left(n,w\right)=-14.60159-2.21277n+180.25742w-0.05482n^{2}+6.51059nw-263.28125w^{2}$$

Also for the root-soil composite, the cohesion *c*(*n*, *w*) can be expressed as:

(9) $$c(n,w)=175.62729-4.03982n-911.83792w-0.29966n^{2}+13.17797nw+1226.56250w^{2}$$

The internal friction angle *φ*(*n*, *w*) can be expressed as:

(10) $$\varphi (n,w)=4.21460-0.31129n+83.89352w-0.18180n^{2}+5.21398nw-158.59372w^{2}$$

**Figure 9:**
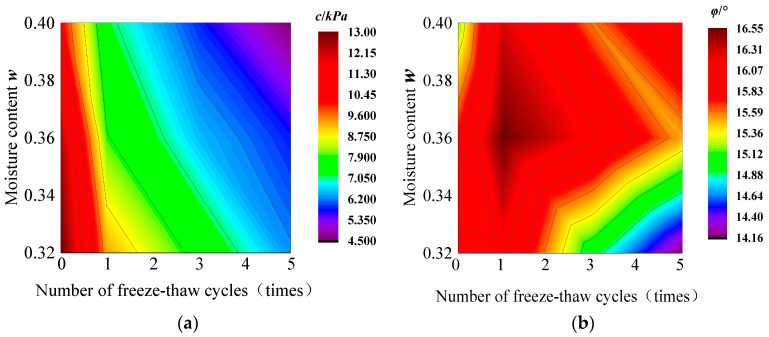
Effect of the number of freeze–thaw cycles and initial moisture content on the cohesion (**a**) *c* and internal friction angle (**b**) *φ* of bare soil.

**Figure 10:**
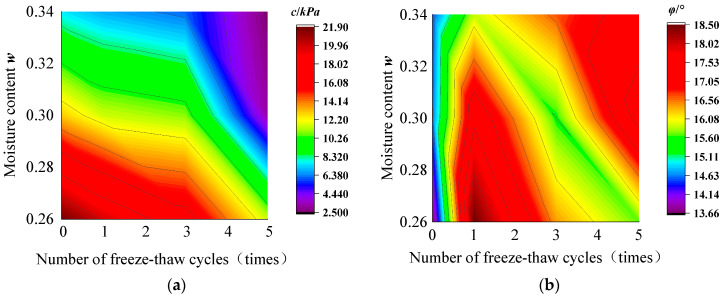
Effect of the number of freeze–thaw cycles and initial moisture content on cohesion (**a**) *c* and internal friction angle (**b**) *φ* of root-soil composite.

Based on the results of the direct shear test, the contour clouds of the effects of the number of freeze–thaw cycles, *n*, and the initial moisture content, *w* on the cohesion *c* and the internal friction angle *φ* of the bare soil are fitted in Figure 9a and Figure 9b, respectively. The contour clouds of the effects on the cohesion, *c*, and the internal friction angle, *φ*, of the root-soil composite are in Figure 10a and Figure 10b, respectively.

### 3.5. Simulation Calculation Results

#### 3.5.1. Stress Distribution Results

**Figure 11:**
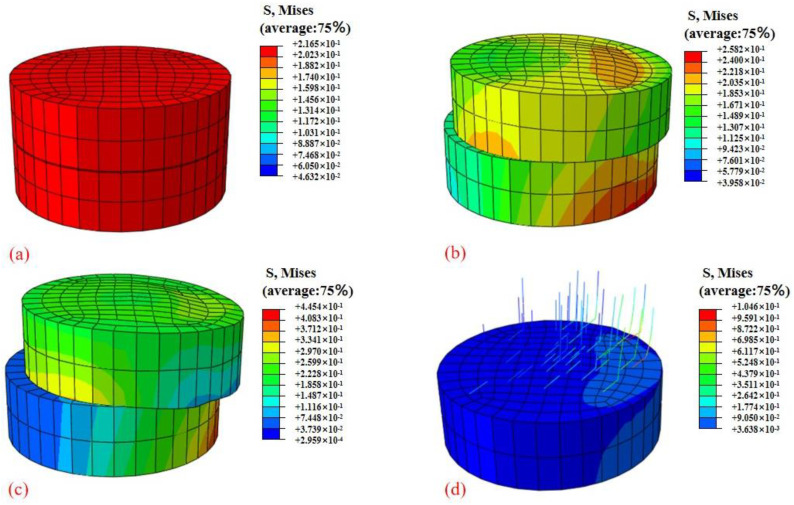
Numerical simulation of root-soil composite with stress states at each time step. (**a**) Adding vertical pressure, (**b**) start of the shear test, (**c**) end of the shear test, and (**d**) stress distribution of root-soil composite after shearing.

To approve the accuracy and applicability of the fitted equations (i.e., Equations (7) and (8)), they were consolidated into the ABAQUS finite element software to define the material properties for the direct shear test simulation. By doing so, the program could simulate and analyze the deformation and stress dispersion of the shear surface within the circular box. This simulation enabled a deeper understanding of the root reinforcement mechanism under freeze–thaw cycles. By observing the behavior of the root-soil composite during the simulation, the viability of the fitted equations in predicting the shear strength and other mechanical properties of the composite under changing freeze–thaw conditions can be assessed, hence providing important insights into the role of roots in soil stabilization. Figure 11 shows the stress distribution of the root-soil composite shear box in the early stage of shearing. At the beginning of the simulation, when the soil sample has not yet been sheared, the soil is compressed due to the vertical pressure on the upper surface. The soil stress distribution is more uniform and the stress is smaller as shown in Figure 11a. With the continuous movement of the upper box, the shear force on the shear surface increases continuously as shown in Figure 11b, until a shear band with a longitudinal width of approximately 6 mm is formed at the shear part. When the upper and lower boxes of soil gradually staggered, the shear progress stopped as shown in Figure 11c. Figure 11d shows the stress of the root-soil composite after the end of the shear. In the direct shear test after freeze–thaw cycles, the stress value at the roots was notably higher than that of the surrounding soil.

#### 3.5.2. Comparison of Shear Stress–Displacement Curves

**Figure 12:**
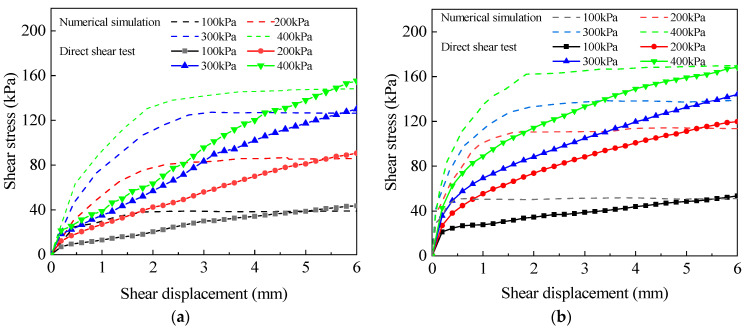
Comparison of stress–displacement paths between direct shear test and numerical simulation of (**a**) bare soil and (**b**) root-soil composite.

To mitigate stress concentration caused by the non-uniform distribution of stresses over different parts of the soil shear surface during the shear process, an approach was utilized to extricate the average value of stresses at each time step of the shear surface. By considering the average stress values, the risk of localized stress concentrations was reduced, providing a more representative and stable measurement of shear stress during the soil shear process. Then, the displacement changes at each time step of the shear zone were extracted to obtain the shear stress–displacement curves under four types of vertical pressures, which were shown as dashed lines in Figure 12. Figure 12a,b show the experimental and numerical stress–displacement curves of bare soil with *w*_i_ = 32% and root-soil composite with *w*_i_ = 26% after one freeze–thaw cycle for the direct shear test and simulation, respectively.

**Figure 13:**
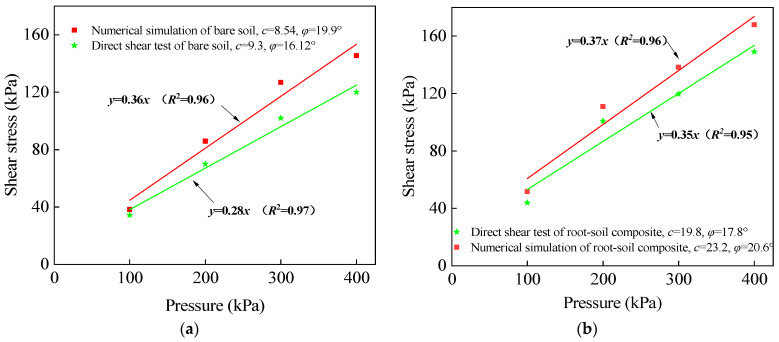
Comparison of the direct shear test of (**a**) bare soil and (**b**) root-soil composite with numerical simulation *c* and *φ*.

To analyze the shear stress of the two types of curves at a shear displacement of 4 mm and under four different vertical loads, a linear fitting approach was utilized. The shear stress–vertical pressure curves for both uncovered soil and root-soil composite were plotted in Figure 13a and Figure 13b, respectively. The fitted data enable comprehensive comparison of the shear stress behavior between the two materials under varying vertical pressures. The fitting results show that the difference between the numerical simulation and the direct shear test is 8.2% and 17.2% for the cohesion of bare soil and root-soil composite, respectively, and 23.4% and 15.7% for the internal friction angle, respectively.

## 4. Discussion

### 4.1. Effect of Initial Moisture Content on the Shear Strength of Specimens under Freezing and Thawing

During the freeze–thaw cycle, the original stability of the soil structure will be damaged due to the continuous change in the interface bearing capacity. The freeze–thaw cycle induces changes in the physicochemical properties of the soil. During this cycle, inside water moves, leading to ice binding and melting, which subsequently changes the interconnection and arrangement of soil particles. As a result of these changes, the soil porosity increases, contributing to modifications in its overall structure. Even though the ice melts into the water later, it is difficult to return to the original pore state. Therefore, the first freeze–thaw significantly reduces the shear strength of the bare soil.

Compared with bare soil, the reduction value and the shear strength of root-soil composites are reduced. Throughout the freeze–thaw cycle, the presence of roots mitigated the weakening impact on the soil’s shear strength. This change brought about the previously weaker bare soil procuring bendable properties and showing higher strength as a ductile material, essentially attributed to the strengthening impact of the roots.

### 4.2. Effect of the Number of Freeze–Thaw Cycles on the Shear Strength of Specimens

The initial freeze–thaw cycle had the most significant effect on the cohesion of the uncovered soil and its shear strength [32]. This initial freeze–thaw cycle caused the most noticeable changes in these properties compared to subsequent freeze–thaw cycles.

Previous research reported that soil strength reduction during the freeze–thaw cycles mainly occurred in the first to seventh cycles. Our preliminary studies showed that the change is not obvious after five freeze–thaw cycles. Therefore, the number of freeze–thaw cycles is five in this paper, but the decreasing trend of soil shear strength is in agreement with the findings of other researchers [33,34]. For bare soil in Figure 6a, the percentage of decrease gradually tends to level off when experiencing 3 to 5 freeze–thaw cycles, which is consistent with the previous research [35]. In Figure 6b, the internal friction angle, *φ*, does not change significantly and regularly with the increasing number of freeze–thaw cycles for bare soils with different moisture contents. That means *φ* does not change significantly under the freeze–thaw cycles.

The cohesion observed in the root-soil composite is attributed to the attachment between soil particles and between soil particles and the roots. The significant factor that recognizes the behavior of the bare soil from the root-soil composite is the presence of roots, which remain in an active state during the initial freeze–thaw cycle. This active state of the roots significantly prevents the development of water particles during the ice-water stage change, subsequently slowing down the increment in soil porosity throughout the freeze–thaw process [36]. As a result, the root-soil composite exhibits higher ductility to begin with three freeze–thaw cycles compared to the bare soil, leading to a decreased freeze–thaw effect on cohesion. After three freeze–thaw cycles, the decreasing cohesion of the root-soil composite increased. This is because the roots were inactivated under repeated freezing and thawing, resulting in the reduction in the entire shear strength of the root-soil composite, and then the cohesion is reduced.

### 4.3. Influence of Root Content on the Shear Strength of Root-Soil Composite during the Freeze–Thaw Cycle

The mentioned discoveries assist in highlighting the considerable role of roots in inhibiting the increment of soil porosity during the freeze–thaw cycles. With the same initial moisture content and freeze–thaw cycles, a clear trend developed: the lower the root content, the more prominent the reduction in the cohesion of the root-soil composite during the freeze–thaw cycles. Subsequently, this leads to a more articulated impact on soil stability.

### 4.4. Numerical Simulation Discussion and Mechanism Analysis

There is substantial friction at the root–soil interface, and the roots themselves contribute to a tensile effect during the test. As a result, the roots play a significant role in redistributing and sharing the shear stress in soil interfacing, leading to an enhancement in the overall shear strength of the soil. This phenomenon highlights the significant reinforcing impact of roots in improving the stability and mechanical properties of the root-soil composite under freeze–thaw conditions.

In the numerical stress–strain curves, the shear stress first increased gradually and then stabilized. Under the same vertical pressure, the entire shear stress of the numerical simulation is smaller compared with that of the direct shear test. The initial conditions of the numerical simulation are strictly controlled, while the parameters and boundary conditions in the test are random and difficult to accurately obtain. There are unavoidable errors while within the acceptable scope of engineering.

## 5. Conclusions

This paper took the subalpine meadow soil in the Kangding area of west Sichuan, China as the research object. The freeze–thaw cycle test, the direct shear test, and numerical simulation analysis were used to investigate the bare soil and undisturbed root-soil composite. Strength parameters such as cohesion and internal friction angle were quantitatively analyzed. The following conclusions were obtained:

(1) The first freeze–thaw cycle caused the largest impact on the shear strength of the bare soil. After 3–5 freeze–thaw cycles, soil shear strength stabilized. The weakening effect on the root-soil composite is mitigated during the two early freeze–thaw cycles. Roots play an inhibitory role in the increase in soil porosity during freeze–thaw cycles.
(2) The percentage decrease in cohesion for both bare soil and root-soil composite gradually becomes more pronounced as the moisture content increases. The increasing root content can nonlinearly inhibit the decrease in the cohesion and shear strength. The freeze–thaw cycle mainly affects the change in the cohesion of bare soil and root-soil composite rather than the change in *φ* and then affects their shear strength.
(3) The fitting formulas applied to the numerical simulation of the direct shear test under freezing and thawing of the soil were feasible and accurate. For bare soil, the differences in cohesion and internal friction angle between the numerical simulation and the direct shear test are 8.2% and 23.4%, respectively. For root-soil composite, those differences are 17.2% and 15.7%, respectively.

## Figures and Tables

**Table 1:** Basic physical properties of soil.

Geotechnical Types	Density/ (g·cm^−3^)	The Natural Moisture Content/%	Particle Composition/%		
			>10 mm	1~10 mm	<1 mm
Root-soil composite	1.018	32	/	/	/
Bare soil	1.249	40	20	50	30

**Table 2:** Design of the freeze–thaw cycle direct shear test.

Experimental Soil	Initial Moisture Content/%	Freezing Temperature/°C	Melting Temperature/°C	Number of Freeze–Thaw Cycles
Root-soil composite	26	−10	20	0, 1, 3, 5
	30	−10	20	0, 1, 3, 5
	34	−10	20	0, 1, 3, 5
Bare soil	32	−10	20	0, 1, 3, 5
	36	−10	20	0, 1, 3, 5
	40	−10	20	0, 1, 3, 5

The site survey found that the roots can absorb water, which results in the water content of the root-soil composite being lower than that of the bare soil. Therefore, the moisture content of samples was set according to the actual field conditions.

## Data Availability

Data are contained within the article.
